# Non-celiac Enteropathy and Olmesartan: An Essential Consideration

**DOI:** 10.7759/cureus.54373

**Published:** 2024-02-17

**Authors:** Sotirios G Doukas, Panagiotis G Doukas, Sugirdhana Velpari

**Affiliations:** 1 Department of Medicine, Section of Gastroenterology and Hepatology, Rutgers-Robert Wood Johnson Medical School/Saint Peter's University Hospital, New Brunswick, USA; 2 Department of Medicine, Rutgers-Robert Wood Johnson Medical School/Saint Peter's University Hospital, New Brunswick, USA

**Keywords:** duodenal biopsy in malabsorption syndrome, enteropathy, non-celiac, sprue-like enteropathy, olmesartan-induced enteropathy

## Abstract

Emerging evidence has shed light on non-celiac causes of enteropathy in recent years, presenting a diagnostic challenge for clinicians. This study discusses the diagnostic challenges related to non-celiac enteropathy, specifically focusing on olmesartan-induced enteropathy (OIE). A 73-year-old lady presented to the emergency department with a six-month history of watery diarrhea exacerbated by food intake and significant weight loss. The patient at admission was found to be dehydrated with severe hypokalemia and hypocalcemia. The extensive testing that was performed was unremarkable, including celiac disease panel, enteric panel, ova and parasites, *Clostridium difficile*, fecal calprotectin, and computed tomography of the abdomen and pelvis. A significant electrolyte imbalance was corrected at admission, and subsequent upper endoscopy investigation with duodenal biopsies revealed moderate to severe villi blunting with a significant intraepithelial infiltrate of CD3+ lymphocytes. A colonoscopy that was performed at the same time was unremarkable, with negative biopsies for microscopic colitis. Given the suspicion of OIE, olmesartan was discontinued. One-month follow-up revealed resolution of malabsorption, with electrolyte normalization and duodenal biopsies showing improved duodenitis. This study emphasizes the importance of considering medication history and ruling out other potential causes of enteropathy. Olmesartan is an angiotensin II receptor antagonist that is commonly prescribed for hypertension. However, in rare cases, it may induce enteropathy, which often remains underdiagnosed. This rare side effect may present as chronic diarrhea, weight loss, and signs of malabsorption. Interestingly, OIE presents with overlapping clinical and histopathological features to celiac disease and, therefore, may mislead physicians to an extensive diagnostic investigation. Greater awareness of medication-related diarrheal syndromes such as OIE should be promoted, given that simple discontinuation of the medication can lead to dramatic clinical improvement.

## Introduction

Enteropathy, characterized by chronic diarrhea and villous atrophy, is commonly associated with celiac disease, an immune-mediated disorder triggered by gluten ingestion [1]. However, emerging evidence has shed light on non-celiac causes of enteropathy in recent years, presenting a diagnostic challenge for clinicians. One such cause is olmesartan-induced enteropathy (OIE), a condition associated with the long-term use of the angiotensin receptor blocker olmesartan medoxomil [2,3].

The diagnosis of non-celiac enteropathy mandates thorough consideration of a broad spectrum of differentials. While celiac disease remains the primary consideration, other etiologies, such as medication-induced enteropathy, infections, inflammatory bowel disease, and autoimmune disorders, need to be evaluated [3,4]. The clinical presentation and histopathological findings can overlap between these entities, making clinical diagnosis essential for appropriate management.

This study discusses the challenges in diagnosing non-celiac enteropathy, specifically focusing on OIE, by presenting a relative case and performing a pertinent literature article review. The knowledge gained from understanding the diverse differentials and diagnostic hurdles will aid in improving patient outcomes and preventing unnecessary interventions.

## Case presentation

A 73-year-old woman with a large volume of watery diarrhea for about six months was presented to the emergency department with a history of severe hypokalemia, hypocalcemia, and hypomagnesemia. She reported daily occurrences of large, non-bloody bowel movements exacerbated by food intake. Abdominal symptoms included excessive borborygmi and mild colic-like abdominal pain, nausea, vomiting, fever, or melena. Despite a general diet, she reported remarkable weight loss over the same period and endorsed no recent travel or sick contacts. Previous evaluations two months ago, including esophagogastroduodenoscopy (EGD) and colonoscopy, were unrevealing, but biopsies were not obtained from either the duodenum or the colon. Concurrent outpatient tests for celiac disease panel, enteric panel, ova, parasites, *Clostridium difficile*, and fecal calprotectin yielded no significant findings. Computed tomography (CT) of the abdomen and pelvis with intravenous (IV) and oral contrast that was performed at admission showed no acute findings, except mild intra- and extrahepatic biliary duct dilation and atrophy in the tail of the pancreas with a prominent pancreatic duct. The patient has been taking olmesartan 40 mg for more than five years, along with amlodipine 10 mg and doxazosin 2 mg daily, for hypertension control. Other medications include ferrous sulfate, prescribed after a recent diagnosis of iron-deficient anemia, atorvastatin 40 mg for hyperlipidemia, ibandronate 150 mg monthly for osteoporosis, and omeprazole 40 mg for gastroesophageal reflux. Her past medical history was significant for fractures and hernia repair. She neither used alcohol, tobacco, nor illicit substances. The patient has no family history of inflammatory bowel disease or malignancy.

**Figure 1:**
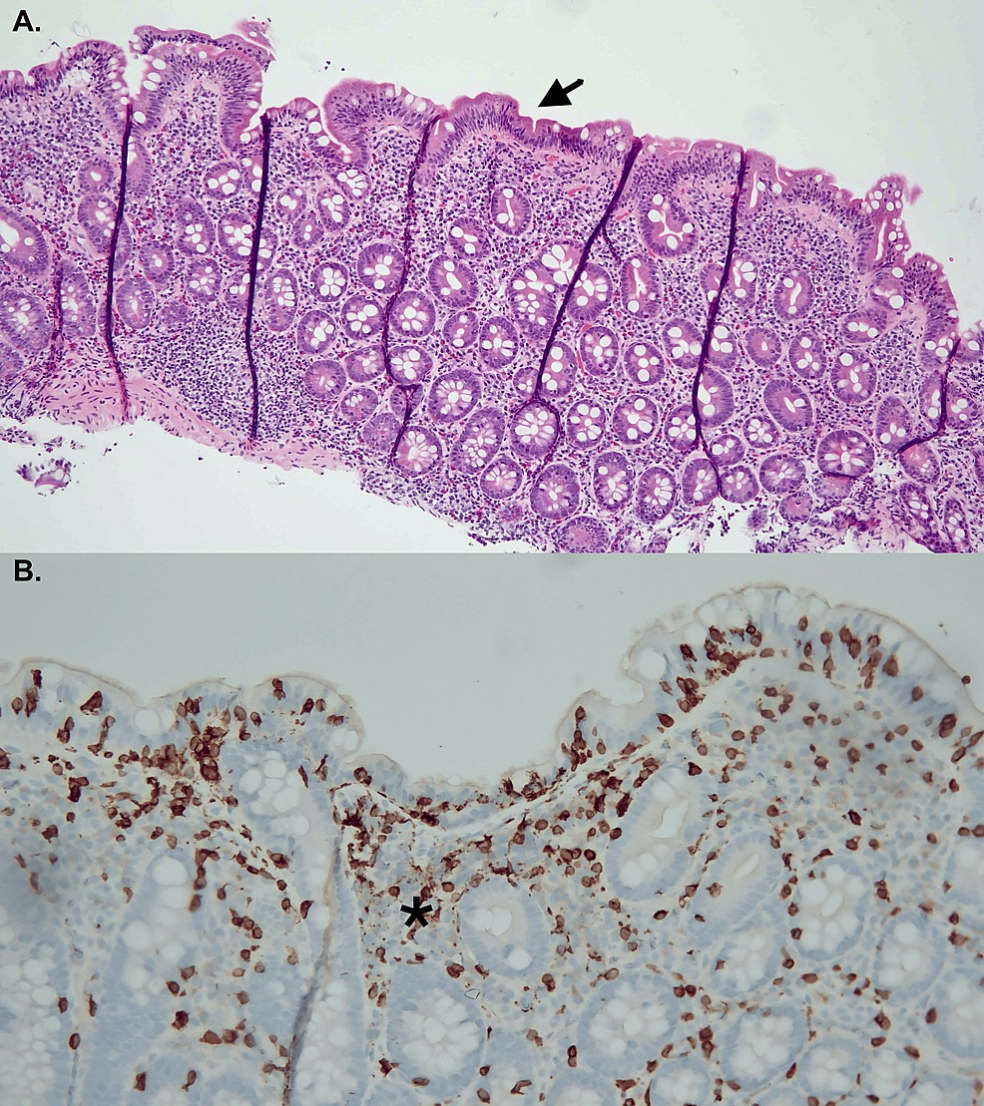
Duodenal histology at the index endoscopy (A) Hematoxylin and Eosin stain showing severe villous flattening (arrow). (B) CD3+ immunostaining shows increased intraepithelial lymphocytes (star).

At presentation, the patient had potassium of 2.5 mmol/L, calcium of 5.9 mmol/L (6.9 mmol/L after corrected for albumin), albumin of 2.8 mg/dl, and bicarbonate of 17 mmol/L with chloride of 112 mmol/L, sodium of 141 mmol/L, and an anion gap of 12. Liver enzymes and total bilirubin were within the normal range. Except for a hemoglobin level of 12.5 g/dL, the patient’s complete blood count was normal. Stool enteric polymerase chain reaction panel (PCR), stool ova and parasites, and *Clostridium difficile* PCR were negative. A detailed description of the laboratory investigations is presented in Table 1. After proper resuscitation and repletion of electrolytes, the patient underwent an EGD that showed normal gastric mucosa and duodenitis with villi blunting. Duodenal biopsies showed moderate to severe villi blunting with a significant intraepithelial infiltrate of a cluster of differentiation-3-positive (CD3+) lymphocytes (Figure 1). Acid-fast stain and Gömöri's methenamine silver stain were negative, suggesting against *Mycobacterium avium* complex and fungal infection, respectively. The histologic analysis performed by the pathologist suggested a malabsorption pattern of injury with differentials including medication-induced injury, gluten-sensitive enteropathy, tropical sprue, chronic malnutrition, and immune-related disorders. A colonoscopy was also performed at that time, which was unremarkable, with random colonic biopsies negative for microscopic colitis.

**Table 1:** Laboratory values and microbiology assays ^a^Reference ranges may be affected by many variables, including the patient population and the laboratory methods used. The reference values used at Saint Peter’s University Hospital in New Brunswick are for adults who have no medical conditions that could affect the results.

Parameters	On admission	Reference range, adults^a^
Hemoglobin (g/dL)	12.5	12.0-16.0
Hematocrit (%)	36.1	35.0-47.0
Mean corpuscular volume (fL)	90.3	80-100
mean corpuscular hemoglobin (pg)	31.3	27.0-34.0
Mean corpuscular hemoglobin concentration (g/L)	34.6	28.0-37.1
White-cell count (per mm)	4.9	4.0-11.0
Red blood cells (10^6^/cumm)	4.00	3.80-5.20
Neutrophils (%)	85.0	37.0-75.0
Lymphocytes (%)	8.7	12.0-50.0
Monocytes (%)	5.7	0.0-10.0
Eosinophils (%)	0.4	0.0-7.0
Platelet count (10^3^/cumm)	187	150-400
Prothrombin time (sec)	16.1	10.4-13.7
International normalized ratio	1.39	0.89-1.11
Partial thromboplastin time (sec)	30.3	27.2-35.7
Total protein (g/dL)	5.3	6.0-8.0
Albumin (g/dL)	2.8	3.2-4.6
Urea nitrogen (mg/dL)	22	9-28
Creatinine (mg/dL)	0.90	0.52-1.04
Estimated glomerular filtration rate (mL/min/1.73m^2^)	74	>60
Calcium (mg/dL)	5.9	8.4-10.0
Glucose (mg/dL)	112	82-115
Sodium (mmol/L)	127	53-141
Potassium (mmol/L)	2.5	3.5-5.1
Chloride (mmol/L)	112	21-33
HCO3 (mmol/L)	17	21-33
Anion gap	12	
Total bilirubin (mg/dL)	0.3	0.1-1.2
Alkaline phosphatase (mg/dL, U/L)	127	53-141
Aspartate transaminase (U/L)	33	14-36
Alanine transaminase (U/L)	24	0-35
Phosphorus (mg/dL)	2.6	2.8-4.1
Magnesium (mg/dL)	0.70	1.60-2.66
Iron (mcg/dL)	38	50-170
Direct iron bind capacity (mcg/dL)	173	264-497
Saturation (%)	22	15-50
Vitamin B12 (pg/mL)	>1,000.0	180.0-914.0
Ferritin (ng/mL)	627.0	11.0-164.0
Folate serum (ng/ml)	11.7	>2.8
Osmolarity (mOsm/kg of water)	294	280-290
HIV 4^th^ generation test	Negative	-
*Clostridium difficile* toxin by polymerase chain reaction	Negative	-
Ova/parasite – cryptosporidium/giardiasis stool Ag	Negative	-
Anti-tissue transglutaminase IgA (U/mL)	<1.0	-
Fecal fat, qualitative (Sudan III stain)	Abnormal	-

Given that repeat tissue transglutaminase and endomysial immunoglobulin A (IgA) antibodies were negative with normal IgA levels (93 mg/dl), the diagnosis of OIE was considered, and the medication was discontinued. Initially, during the patient's hospital stay, cholestyramine 4 mg daily and loperamide were administered in an attempt to control her symptoms. Later, due to the suspicion of secondary chronic pancreatitis based on imaging findings and abnormal fecal fat testing, cholestyramine was stopped, and pancreatic enzyme supplementation was initiated along with loperamide 2 mg two times per day, as needed. After the patient’s symptoms were controlled, she was discharged with a recommendation to follow up closely as an outpatient.

**Figure 2:**
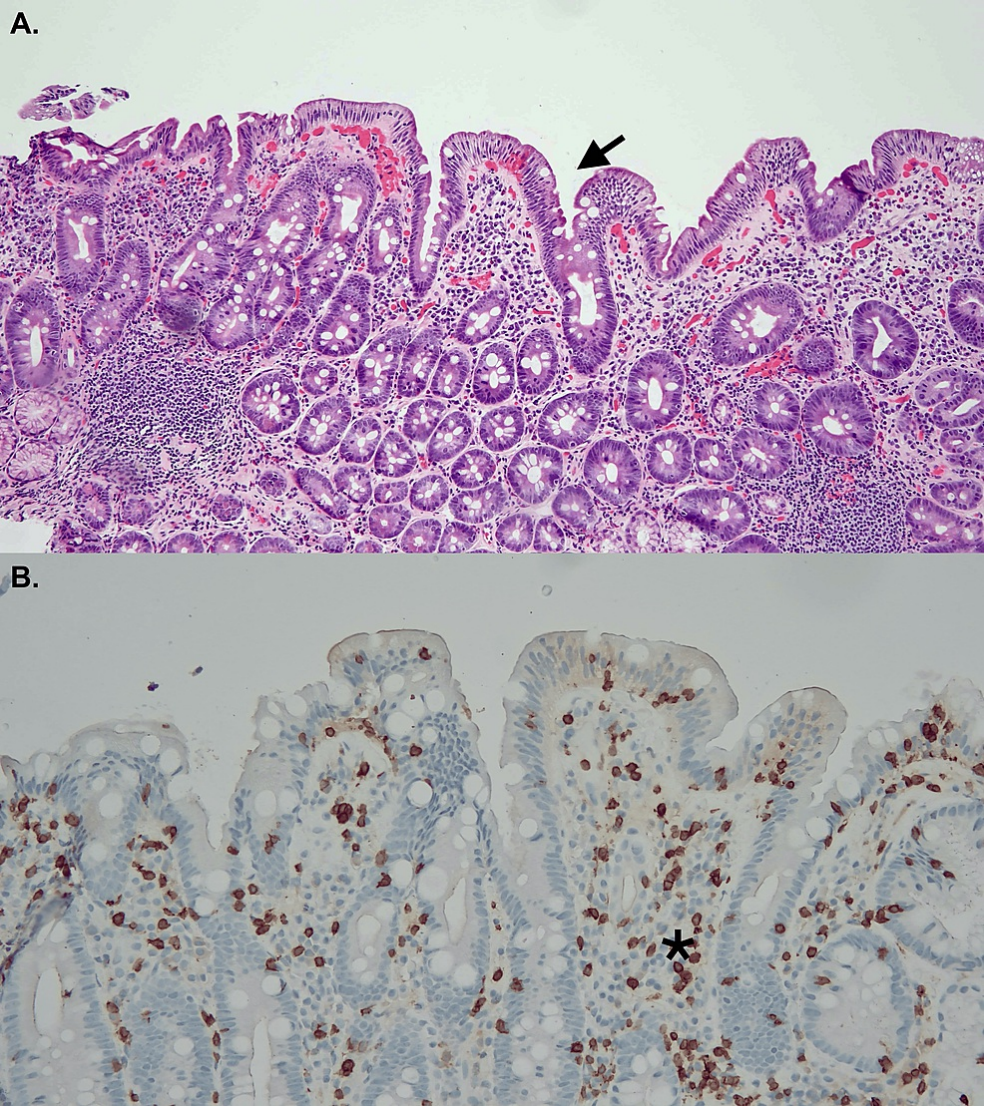
Duodenal histology at follow-up biopsy (A) Hematoxylin and eosin stain significantly less villous flattening (arrow). (B) CD3+ immunostaining showed decreased intraepithelial lymphocytes (star).

After one month, the patient underwent endoscopic ultrasonography of the pancreas and biliary system, which showed evidence suggestive of chronic pancreatitis and a mildly dilated biliary system without obstruction. Endoscopic evaluation at this point showed improved duodenitis, with biopsies showing less blunting and no CD3+ lymphocytes (Figure 2). Although we initially planned to perform additional stains, such as periodic acid-Schiff (to rule out Whipple's) and Congo red stain (to rule out amyloidosis), during the repeat endoscopic evaluation, given the dramatic clinical and histologic improvement at the follow-up, it was considered unnecessary and no additional stains were performed. The patient reported that her diarrhea resolved completely after two weeks of discharge, and her weight improved from 43.2 kg to 55 kg within a month. The baseline weight prior to enteropathy was 52.1 kg. Serum electrolytes were normalized entirely, too. After discontinuation of olmesartan, blood pressure remained controlled with the rest of the home medication, amlodipine 10 mg and doxazosin 2 mg daily. The patient remained asymptomatic with no further hospitalization for six months after the discontinuation of olmesartan and continued to follow up in the clinic. The patient has given informed consent to document the presented case report.

## Discussion

OIE is a rare type of sprue-like enteropathy associated with the long-term use of the angiotensin receptor blocker olmesartan medoxomil [3]. Although the Food and Drug Administration has reported enteropathy as a possible side effect of prolonged olmesartan use, the diagnosis of OIE in clinical practice may prove challenging [3,5]. This case report and literature review will help contribute to the understanding of OIE by presenting the recommended diagnostics and recommended management.

The patient in this case report presented with chronic diarrhea, weight loss, and electrolyte abnormalities, including severe hypokalemia, hypocalcemia, and hypomagnesemia, highly suggestive of malabsorption. Also, the patient's low albumin levels and abnormal fecal fat test suggested possible protein-wasting enteropathy. After ruling out common infectious causes, including giardia, endoscopic evaluation was performed to obtain duodenal biopsies for enteropathy evaluation. In our case, EGD revealed duodenitis, with biopsies showing blunting of the villi and a significant infiltrate of intraepithelial lymphocytes (IELs), suggesting sprue-like enteritis. In cases of duodenal biopsies suggestive of sprue enteropathy, celiac disease serologies are highly recommended since celiac remains the most common etiology [6].

Although the patient had negative celiac panel results, seronegative disease has been reported in up to 15% of patients with villi flattening [5,7]. So far, there are no known specific histopathologic findings for OIE, with recent studies suggesting a spectrum of the disease with various histopathologic and clinical features. Specifically, mild villus blunting and IEL infiltration might precede typical enteropathy symptoms [8]. Interestingly, a retrospective study showed that up to 50% of patients taking olmesartan might develop at least one sprue-like histologic feature, with other studies suggesting that OIE enteropathy may develop slowly with the insidious onset of symptoms after years of use [9]. Although the exact mechanism involved in the development and progression of OIE is unclear, an immune-mediated disorder has been suggested [3]. The lag time between the initiation of the medication and the onset of symptoms suggests a cell-mediated immune response involved in the process [3,10].

Overall, OIE management involves discontinuing olmesartan and providing proper supportive care. In this case, after olmesartan discontinuation, the patient showed significant improvement in duodenitis and complete resolution of diarrhea. Although so far there are no specific guidelines for symptomatic management, the initiation of a low-fermentable oligosaccharides, disaccharides, monosaccharides, and polyols (FODMAP) diet and loperamide is a reasonable initial approach. Previous studies have shown that pancreatic enzyme supplementation should be considered in patients with proven celiac disease-induced pancreatic insufficiency [11]. In our case, although the exocrine function of the pancreas was not assessed, given evidence of chronic pancreatitis on the CT scan and signs of malabsorption, pancreatic enzyme supplementation was initiated at presentation. Also, given the high volume of diarrhea, electrolyte abnormalities and dehydration are common findings in these patients. Close monitoring is highly recommended in patients with high-volume diarrhea, which can lead to potential life-threatening electrolyte abnormalities.

Given no specific histopathologic criteria to differentiate OIE from other causes of enteropathies such as tropical sprue, Crohn’s, autoimmune enteropathy, or another medication-induced sprue-like enteropathy, improvement of clinicopathological features after discontinuation of olmesartan is required for a definitive diagnosis. The recovery timeline after olmesartan discontinuation is not clear but overall considered shorter compared to celiac, with symptomatic improvement weeks after and histologic resolution on average eight months after discontinuation [3,12]. In our case, after olmesartan was discontinued, the patient’s diarrhea and electrolyte imbalance resolved within two weeks, and the patient started gaining weight in less than a month. The follow-up duodenal biopsies four weeks after the procedure showed histologic improvement, confirming the diagnosis of OIE. Although colonic biopsies were negative for microscopic colitis in the presented case, colonic biopsies suggestive of microscopic colitis have also been seen in patients with OIE, with previous studies suggesting resolution after discontinuation of olmesartan, and thus should always be done as part of the diagnostic workup [13].

Reinitiating olmesartan has been shown to cause a recurrence of OIE; therefore, it is contraindicated. Although other medications belonging to the angiotensin receptor blocker (ARB) class have been associated with similar side effects, though less frequent than olmesartan, it is not unreasonable to consider switching to a different ARB for management. [12] However, this is not a priority; therefore, an agent from another class may be a better option to avoid a class effect altogether.

The reported case highlights the importance of considering non-celiac causes of enteropathy, such as OIE, in patients presenting with chronic diarrhea and villous atrophy. Accurate diagnosis is crucial to guide appropriate management, avoid unnecessary interventions, and prevent potential complications associated with prolonged use of olmesartan. It is worth noting that distinguishing OIE from celiac disease can be challenging due to overlapping clinical and histopathological features. This emphasizes the importance of considering medication history, performing thorough diagnostic evaluations, and ruling out other potential causes of enteropathy. Last, we would like to acknowledge the valuable contribution and crucial input of the radiology and pathology teams in this investigation.

## Conclusions

This case report and literature review contribute to the existing knowledge of OIE. The presented case underscores the significance of considering OIE in the differential diagnosis of non-celiac causes of enteropathy, especially in patients on long-term olmesartan therapy. Early recognition, prompt medication discontinuation, and supportive care can significantly improve clinical outcomes. Further research is warranted to better understand the pathophysiology and create optimal strategies to manage this disease.
